# Characteristics of forensic psychiatric patients with a neurocognitive disorder

**DOI:** 10.1192/bjo.2024.712

**Published:** 2024-05-20

**Authors:** Jesse Meijers, Niki C. Kuin, Erik J. A. Scherder, Joke M. Harte

**Affiliations:** Willem Pompe Institute for Criminal Law and Criminology, Utrecht University, The Netherlands; and Judicial Complex Zaanstad, Dutch Custodial Institutions Agency, Ministry of Justice and Security, The Hague, The Netherlands; Penitentiary Institution Vught, Dutch Custodial Institutions Agency, Ministry of Justice and Security, The Hague, The Netherlands; and Pieter Baan Centre, Netherlands Institute of Forensic Psychiatry and Psychology, Ministry of Justice and Security, The Hague, The Netherlands; Section of Clinical Neuropsychology, Faculty of Behavioural and Movement Sciences, Vrije Universiteit Amsterdam, The Netherlands; Department of Criminology, Faculty of Law, Vrije Universiteit Amsterdam, The Netherlands

**Keywords:** Prison, forensic psychiatry, neurocognitive disorders, neuropathology, dementias/neurodegenerative diseases

## Abstract

**Background:**

Emotional and behavioural disturbances accompanying neurocognitive disorders may sometimes lead to a criminal offence. Our knowledge of this specific forensic subpopulation is lagging behind the knowledge on, and attention for, ‘classic’ psychiatric disorders in forensic populations.

**Aims:**

To gain knowledge on the prevalence and characteristics of individuals with neurocognitive disorders in the forensic population.

**Method:**

This retrospective database study uses an anonymised data-set of the National Database of penitentiary psychiatric centres (PPC) (*N* = 8391), which collects data on all patients admitted to one of the four PPCs (mental health clinics within the prison system) in The Netherlands since May 2013. Inclusion criterion for this study was the presence of a *Diagnostic and Statistical Manual of Mental Disorders*, fourth edition, text revision (DSM-IV-TR) or *Diagnostic and Statistical Manual of Mental Disorders*, fifth edition (DSM-5) diagnostic code belonging to the category of neurocognitive disorders.

**Results:**

A DSM-IV-TR or DSM-5 diagnostic code of a neurocognitive disorder was classified in 254 out of 8391 unique individuals, resulting in a prevalence of 3.0% in the total PPC population. The most prevalent diagnosis was unspecified neurocognitive disorder (59.1%). The neurocognitive disorder group significantly differed from a random control group from the database (*n* = 762) on demographic, clinical and criminological variables.

**Conclusions:**

The prevalence of neurocognitive disorders in this real-world clinical sample is remarkably lower than in two earlier studies in similar populations. Also remarkable is the relatively high prevalence of an unspecified neurocognitive disorder. These findings lead us to hypothesise that neurocognitive disorders may be underdiagnosed in this population. Forensic psychiatric settings should evaluate whether they have sufficient expertise available in neuropsychological assessment.

Neurocognitive disorders are a category of disorders of which the core feature is impaired cognitive functions in one or more cognitive domains, i.e. complex attention, executive function, learning and memory, language, perceptual motor control and social cognition.^1,2^ These cognitive impairments are acquired, rather than innate, and may be caused by, for example, traumatic brain injury (TBI), substance misuse or neurodegenerative diseases. The cognitive impairments are often accompanied by behavioural and emotional impairments, such as impulsivity and emotional instability. In certain cases, such impairments may also include aggressive and violent behaviour or other types of adverse or criminal behaviours, such as fire setting, stealing or sexual offenses.^3,4^

## Neuropathology, neurocognitive disorders and criminal behaviour

Direct evidence for the relationship between neuropathology, neurocognitive disorders and criminal behaviour comes from longitudinal studies. One longitudinal birth cohort study found that TBI sustained between the ages of 0 and 21 years led to an increased risk of arrest, with early substance misuse as an important mediating factor for those who sustained TBI between the ages of 0 and 5 years.^5^ In a systematic review of 16 studies on this subject, the authors concluded that both cross-sectional as well as longitudinal studies support the associations between childhood TBI and antisocial and criminal behaviour.^6^ Furthermore, compared with offenders without TBI, offenders with TBI show more aggression and delinquent behaviour.^7,8^ The relationship between dementia and increased criminal behaviour has been well established. Dementia can be related to an increased risk for a variety of offenses, such as violence, stealing, fire setting or inappropriate sexual behaviour.^3,4,9^ A recent nationwide register study from Finland, using data on more than 92 000 patients with dementia, showed a significantly higher risk of committing a crime in the 4 years before the patients were actually diagnosed with dementia.^10^ Interestingly, using the same data, the researchers found a lower risk of criminal behaviour after receiving a diagnosis. This can most probably be ascribed to factors such as progression of the disease, a high mortality rate and the protective effect of the treatment a patient usually receives after the diagnosis.^11^

Substantiating the abovementioned findings on the relationship between neurocognitive functions, neuropathology, neurocognitive disorders and criminal behaviour are studies that provide a more indirect form of evidence, such as prevalence studies in prisons and forensic psychiatry. Earlier meta-analyses reported prevalence numbers for TBI of 50–60%,^12,13^ and a more recent meta-analysis reports a prevalence of 46%.^14^ An important caveat is that the studies included in the meta-analyses were highly heterogeneous regarding their diagnostic methods, ranging from asking participants two questions on TBI to the use of more extensive structured instruments.^14,15^ Studies on the prevalence of dementia among prisoners are scarce. A recent review suggests that one in five older prisoners (aged ≥55 years) may meet the criteria for dementia, which is considerably higher than the general population.^16^

## Neurocognitive disorders in forensic psychiatry

Although the prevalence of TBI has been studied quite extensively in prisoners, and attention for dementia in prisoners is increasing,^16,17^ there are only a few studies that focus on the prevalence of neurocognitive disorders in forensic psychiatric settings. Most studies that do so, focus on elderly patients and dementia specifically, making it difficult to establish the prevalence in the forensic psychiatric population as a whole and to gain insight in the broad spectrum of neurocognitive disorders. In a review of seven studies on elderly patients in forensic psychiatric settings, prevalence numbers for dementia, TBI or ‘organic brain syndromes’ (an obsolete term that meant to include dementia and TBI) ranged from 10 to 40%.^18^ One of the studies in this review reported a prevalence of organic brain syndromes of 33% in patients aged ≥60 years, and 12% in patients between the ages of 16 and 59 years.^19^ Other studies that focused on all age groups, rather than the elderly, found a prevalence of 23% for TBI in a Canadian sample,^20^ and 26% for cognitive impairment and 15% for TBI in a New Zealand sample.^21^ Taking another approach, one study investigated computed tomography and magnetic resonance imaging scans of inmates from a high-security forensic institution that were referred because of somatic complaints (e.g. headaches or vertigo). The authors found a significantly higher degree of signs of neuropathology in the forensic population than in healthy non-forensic controls (46% *v.* 8%). Saliently, these patients had previously not been considered to have a neurocognitive disorder.^22^ Finally, in a recent study, 20% of a cohort of 638 men in a high-secure forensic unit in Canada were identified as having acquired brain injury.^23^ The authors found that acquired brain injury was related to greater adverse childhood experiences, perinatal and childhood health problems and a lower socioeconomic status. They were also found to have a higher risk of violence and were more often diagnosed with mood and personality disorders, and less often with schizophrenia spectrum disorders. To our knowledge, no further studies have investigated the prevalence of neurocognitive disorders in forensic psychiatric populations, or the characteristics of this subgroup.

## Psychiatric care in Dutch prisons

In The Netherlands, prisoners (both remand and sentenced prisoners) that require more intensive clinical care for serious mental illness than can be provided in regular regimens may be admitted to one of the four penitentiary psychiatric centres (PPCs). PPCs are mental health clinics within the penitentiary institutions in The Netherlands and are formally still prisons, but may otherwise be most comparable to forensic psychiatric hospitals or similar facilities in other countries. The most notable difference with forensic psychiatric hospitals in The Netherlands or other countries is the judicial status of the patients. Patients retain the status of remand or sentenced prisoner, and are placed in a PPC because of their right to appropriate mental healthcare as a prisoner, i.e. they are not court-ordered to treatment in a PPC. The main goals of the PPCs are diagnosis and treatment of mental illness, with a focus on stabilisation of the psychiatric symptoms and reduction of criminogenic risk factors. Within 24 h after admission, a psychologist and psychiatrist reach a first diagnosis based on their independent consultations, the available medical history and information from the referring party. During their stay in the PPC, further diagnostic methods may be applied based on the individual needs of the patient. Schizophrenia spectrum and other psychotic disorders are the most prevalent in this population (56.7%).^24^ For the majority of the PPC patients, behavioural problems are the primary reason of admission, such as dangerous behaviour toward themselves or others that makes it impossible to provide them with the necessary psychiatric care in a regular regimen. One can therefore expect that prisoners with a neurocognitive disorder would often be referred to a PPC as well, especially when staff observe that these individuals cannot function on a sufficient independent level because of severely impaired cognitive function, or when the disorder is accompanied by severe behavioural disturbances.

To date, however, no studies have investigated the prevalence of neurocognitive disorders in these forensic psychiatric facilities, or the characteristics of this subgroup. As all PPCs in The Netherlands are required to systematically collect data on their patients, this study aims to make use of the opportunity to utilise a real-world clinical data-set to describe the prevalence of neurocognitive disorders in prisoners admitted to a PPC, and to investigate the characteristics of this specific subgroup of patients. We aim to add to the scarce literature on the prevalence of neurocognitive disorders in forensic psychiatric populations, and the characteristics of this specific subgroup.

## Method

### Research design

Since 1 May 2013, the four PPCs in the Netherlands systematically gather information on all prisoners admitted to a PPC, resulting in the National Database PPC. This database contains, among others, diagnostic information, demographic characteristics and criminal records. Primarily, the data are used for policy making and clinical purposes. This implies that the data (including the diagnostic data) were not collected for the purpose of this specific study, i.e. no specific diagnostic procedure was conducted for the purpose of this specific study. Research is, however, a secondary purpose of the database. Under strict conditions, permission can be obtained to conduct research with an anonymised data-set. Based on the data that were collected from May 2013 to April 2022, consisting of 8391 unique individuals, a subset of the data has been provided to the authors to conduct the present study.

### Sample and control group

The inclusion criterion for the study sample was the presence of a *Diagnostic and Statistical Manual of Mental Disorders*, fourth edition, text revision (DSM-IV-TR) or *Diagnostic and Statistical Manual of Mental Disorders*, fifth edition (DSM-5) diagnostic code belonging to the category of neurocognitive disorders. See Appendix A for the full list of the diagnostic codes included in this study. We included all individuals that were admitted within the period of data collection that met this inclusion criterion. This resulted in a data-set with 254 unique individuals.

The inclusion criterion for the control group was the absence of a neurocognitive disorder, and the absence of missing data in the diagnostic variables. We were provided with a random sample extracted from the National Database PPC consisting of 762 unique individuals (i.e. three times the size of the study sample).

Demographic characteristics of the study sample are presented in Table 1 and compared with the characteristics of the control group. Salient characteristics for both groups are that most individuals were men in both groups (91.7% *v.* 92.1%), almost half did not complete more than a primary education (43.0% *v.* 43.1%), more than a quarter were homeless (26.4% *v.* 30.2%) and almost a third had already received mental healthcare during childhood (31.7% *v.* 33.0%). In addition, the groups differed on several demographic variables. The study sample was 11.6 years older on average (*P* < 0.001), and they were significantly more often of Dutch ethnicity (59.6% *v.* 34.4%; *P* < 0.05). In addition, the study sample had a significantly higher average length of stay in the PPC than the control group (205.9 days *v.* 153.4 days; *t*(307,427) = 2.643; *P* = 0.009).

**Table 1:** Demographic characteristics of the study sample and the control group

	Study sample	Control group
Study variable	% (*n*)	% (*n*)
Age, mean (s.d.)***	48.5 (16)	36.9 (11.2)
Gender	*n* = 254	*n* = 758
Male	91.7 (233)	92.1 (698)
Average length of stay in the penitentiary psychiatric centre, days, mean (s.d.)	205.91 (281.29)	154.39 (180.81)
Education	*n* = 200	*n* = 542
No education	6.0 (12)	5.4 (29)
Primary special education	7.5 (15)	5.0 (27)
Primary education	29.5 (59)	32.7 (177)
Secondary special education	2.5 (5)	3.0 (16)
Secondary education	34.5 (69)	33.8 (183)
Post-secondary education	20.0 (40)	20.3 (110)
Ethnicity	*n* = 211	*n* = 634
The Netherlands*	59.7 (126)	34.4 (218)
Morocco	5.7 (12)	9.3 (59)
Surinam	5.7 (12)	6.9 (44)
The Netherlands Antilles	3.8 (8)	5.0 (32)
Turkey*	1.9 (4)	5.0 (32)
Other, non-Western	11.4 (24)	15.6 (99)
Other, Western*	11.8 (25)	23.7 (150)
Housing at admission	*n* = 227	*n* = 659
Homeless	26.4 (60)	30.2 (199)
Home	53.3 (121)	50.5 (333)
Assisted living	11.5 (26)	9.4 (62)
Mental health facility	8.8 (20)	9.0 (59)
Prison	0.0 (0)	0.9 (6)
History of mental healthcare	*n* = 254	*n* = 762
Childhood mental healthcare	31.7 (69)	33.0 (206)
Out-patient care	72.1 (160)	70.8 (447)
Voluntary in-patient care	50.0 (109)	50.4 (317)
Involuntary in-patient care	27.9 (61)	33.3 (210)
Assisted living	32.1 (70)	29.5 (185)

* *P* < 0.05, ***P < 0.001.

### Measures

The National Database PPC provided all measures. All variables are coded by trained psychologists or criminologists based on information from, for example, patient files, criminal records and anamnestic information. Besides demographic and diagnostic variables, one variable that is used in this study is item H08 (history of mental healthcare) from the HKT-R (Dutch: Historisch Klinisch Toekomst-Revised) risk assessment scheme,^25^ which is a risk assessment instrument that is highly similar to the internationally more widely known Historical Clinical Risk Management-20 (HCR-20).

### Procedure

All data were obtained as an anonymised data-set through an employee of the National Database PPC. Although 160 individuals in the study sample were only admitted to a PPC once, 94 individuals were admitted to a PPC more than once in their lifetime, resulting in separate data for each admission and stay. In these cases, the data from their most recent admission in which they were diagnosed with a neurocognitive disorder was used for the analyses. For the control group, the most recent admission was used.

This retrospective study on an anonymised data-set was approved by the Ethical Review Board of the Faculty of Law, Economics and Governance at Utrecht University (approval number 2022–004). Because of the retrospective nature of the study and the use of an anonymous data-set, informed consent was not obtained as it was not required.

### Data analysis

All descriptive statistics were produced with IBM SPSS Statistics for Windows (version 28). Group comparisons were either conducted with chi-squared tests, with a *z*-test to compare column proportions when appropriate, i.e. in the case of multiple categories within a variable (and Bonferroni-adjusted *P*-values). Independent samples *t*-tests were conducted to assess differences in continuous variables. The prevalence rate was manually calculated based on the number of unique individuals in our data-set, divided by the number of unique individuals in the National Database PPC.

## Results

### Prevalence of neurocognitive disorders

A DSM-IV-TR or DSM-5 (during data collection, the DSM-IV-TR was replaced with the DSM-5) diagnostic code of a neurocognitive disorder (as mentioned in Appendix A) was classified in 254 out of 8391 unique individuals, resulting in a 3.0% prevalence of neurocognitive disorders in the total PPC population.

Looking more closely into the various subtypes of neurocognitive disorders that were identified, 69 individuals (27.1%) had a major neurocognitive disorder, divided over the five separate major neurocognitive disorder diagnoses (e.g. major neurocognitive disorder due to vascular disease, etc.). The most prevalent diagnosis was unspecified neurocognitive disorder (59.1%). See Table 2 for a frequency table of the prevalence of all neurocognitive disorders. Note that the DSM-5 allows for separate coding of some, but not all, neurocognitive disorders.^1^ The prevalence table therefore shows a few specific neurocognitive disorders, such as major neurocognitive disorder due to vascular disease, as well as more broad categories, such as major neurocognitive disorder due to ‘ … ’, where the dots can be varying causes for a neurocognitive disorder, from Alzheimer's disease to TBI, that all fall under the same DSM-5 code.

**Table 2:** Prevalence of neurocognitive disorders

Type of neurocognitive disorder	% (*n*)
Unspecified neurocognitive disorder	59.1 (150)
Major neurocognitive disorder due to … with behavioural disturbance	10.2 (26)
Major neurocognitive disorder due to … without behavioural disturbance	8.3 (21)
Personality change due to another medical condition	7.5 (19)
Mild neurocognitive disorder	6.3 (16)
Alcohol-induced major neurocognitive disorder	5.1 (13)
Major neurocognitive disorder due to vascular disease	3.1 (8)
Alcohol-induced mild neurocognitive disorder	2.0 (5)
Substance-induced major neurocognitive disorder	0.4 (1)
Delirium	0.4 (1)

### Changes in diagnoses of neurocognitive disorders during a stay in the PPC

Diagnoses changed between admission and discharge for 88 individuals (34.6%) in the study sample in two opposite ways. First, there were 42 individuals who did not have a neurocognitive disorder diagnosed at admission, but were diagnosed with one during their stay. In most of these cases, individuals were diagnosed with an unspecified neurocognitive disorder (*n* = 27). Other diagnoses were personality change due to another medical condition (*n* = 4), alcohol-induced major neurocognitive disorder (*n* = 3), mild neurocognitive disorder (*n* = 3) and various major neurocognitive disorders (*n* = 5). Second, 46 individuals were diagnosed with a neurocognitive disorder at admission, but not at discharge. In most cases (*n* = 31), this was because of the removal of the unspecified neurocognitive disorder diagnosis at discharge. There were no data in the database on the reasons for these removals.

### Comorbidity

The number of comorbid disorders was significantly higher in the study sample (mean 2.39, s.d. = 1.12) compared with the control group (mean 1.75, s.d. = 0.86; *t*(358, 210) = 8.33; *P* < 0.001). In the study sample, the most frequent disorders after a neurocognitive disorder were substance misuse and addiction disorders (51.2%), personality disorders (23.2%), and schizophrenia spectrum and other psychotic disorders (20.9%). In the control group, there were significantly more individuals with a schizophrenia spectrum disorder (57.5% *v.* 20.9%; *P* < 0.05) and significantly less individuals with a substance misuse or addiction disorder (41.7% *v.* 51.2%; *P* < 0.05). See Table 3 for the frequency distribution of the number of diagnosed disorders, and the prevalence of all disorders.

**Table 3:** Number of classified DSM-5 disorders and prevalence of specific disorders within the study sample with neurocognitive disorders (*n* = 254) and the control group (*n* = 762)

	Sample	Control group
Study variable	% (*n*)	% (*n*)
Number of disorders***		
0	0.0 (0)	1.8 (14)
1	24.0 (61)	42.7 (325)
2	35.0 (89)	38.1 (290)
3	22.4 (57)	13.9 (106)
4	15.0 (38)	3.0 (23)
5	3.1 (8)	0.5 (4)
6	0.4 (1)	0.0 (0)
Comorbid disorders (DSM-5 categories)		
Substance-related and addiction disorders**	51.2 (130)	41.7 (318)
Personality disorders	23.2 (59)	25.3 (193)
Schizophrenia spectrum and other psychotic disorders***	20.9 (53)	57.5 (438)
Other conditions that may be a focus of clinical attention	14.6 (37)	11.7 (89)
Neurodevelopmental disorders	6.7 (17)	10.9 (83)
Depressive disorders	5.5 (14)	4.2 (32)
Trauma- and stressor-related disorders^1^	5.1 (13)	9.7 (74)
Bipolar and related disorders	3.9 (10)	5.2 (40)
Disruptive, impulse control and conduct disorders	2.8 (7)	2.9 (22)
Paraphilic disorders	1.6 (4)	1.4 (11)
Other mental disorders and additional codes	1.2 (3)	1.8 (14)
Anxiety disorders	0.8 (2)	0.9 (7)
Somatic symptom and related disorders	0.8 (2)	0.8 (6)
Obsessive–compulsive and related disorders	0.4 (1)	0.4 (3)
Sexual dysfunctions	0.4 (1)	0.0 (0)
Medication-induced movement disorders and other adverse effects	0.4 (1)	0.0 (0)

DSM-5, *Diagnostic and Statistical Manual of Mental Disorders*, fifth edition.

1 *P* < 0.05, **P < 0.01, ***P < 0.001.

### Criminological characteristics of the sample

The majority of the study sample (69.0%) was suspected of or convicted for a violent crime at admission, compared with 77% of the control group. Similarly, 74.6% of the study sample already had at least one prior conviction for a violent offense, compared with 78.7% of the control group. The study sample included significantly more repeat offenders with ten or more convictions in the past 5 years (29.8% *v.* 17.5%; *P* < 0.05), which is also reflected by the significantly higher number of individuals with a history of an ISD (Dutch: Inrichting Stelselmatige Daders) measure (a judicial measure specifically for repeat offenders). On the other hand, murder was significantly more prevalent in the study sample (10.4% *v.* 5.8%; *P* ≤ 0.05), whereas severe violence was significantly lower (4.8% *v.* 10.2%; *P* ≤ 0.05). There were no significant differences between the groups in judicial status at admission or reasons for admission in the PPC. See Table 4 for detailed criminological characteristics of the sample, including a more specific frequency distribution per type of crime, and comparisons with the control group.

**Table 4:** Criminological characteristics of the study sample and control group

	Study sample	Control group
Study variable	% (*n*)	% (*n*)
History with judicial measures	*n* = 237	*n* = 675
ISD^a^^,^*	16.0 (38)	10.2 (69)
OTS^b^^,^*	8.4 (20)	14.6 (98)
TBS^c^	5.5 (13)	5.3 (36)
PIJ^d^^,^*	2.1 (5)	5.4 (36)
Criminal history	*n* = 242	*n* = 699
First offender	14.5 (35)	12.4 (87)
3–10 convictions in the past 5 years*	21.5 (52)	29.6 (207)
≥10 convictions in the past 5 years*	29.8 (72)	17.5 (122)
Lifetime history of one or more violent offenses	74.6 (182)	78.7 (544)
Judicial status at admission	*n* = 253	*n* = 755
Remand (awaiting trial)	59.1 (150)	69.4 (524)
Prison sentence	23.6 (60)	21.3 (161)
Judicial measure	14.9 (38)	4.4 (51)
Reason for admission besides psychiatric treatment	*n* = 252	*n* = 757
Aggression to others or goods	18.3 (46)	19.6 (148)
Aggression toward self	14.3 (36)	14.7 (111)
Confusion or disturbance of the peace	27.0 (68)	32.9 (249)
Regression	5.2 (13)	2.0 (15)
Type of crime (leading to current imprisonment)	*n* = 251	*n* = 753
Traffic violation, disturbance of the peace or illegal immigrant	4.4 (11)	3.5 (26)
Drug related crime	2.4 (6)	2.3 (17)
Destruction of property	2.4 (6)	2.1 (16)
Property crime (such as theft)*	21.9 (55)	14.5 (109)
Moderate violence or illegal possession of weapons	23.9 (60)	27.1 (204)
Property crime accompanied with violence	5.6 (14)	8.8 (66)
Severe violence*	4.8 (12)	10.2 (77)
Sex offense	3.6 (9)	4.9 (37)
Sex offense against minors	2.4 (6)	2.4 (18)
Manslaughter	13.1 (33)	11.6 (87)
Fire setting with danger to others	5.2 (13)	6.9 (52)
Murder*	10.4 (26)	5.8 (44)

* *P* < 0.05.

a. Dutch: Instelling Stelselmatige Daders, a 2-year judicial measure for habitual offenders.

b. Dutch: Onder Toezichtstelling, a judicial measure placing the parent of a child under supervision.

c. Dutch: Terbeschikkingstelling, a judicial measure of mandatory treatment to reduce reoffending risk.

d. Dutch: Plaatsing in een Inrichting voor Jeugdigen, a judicial measure of mandatory treatment for individuals falling under the juvenile criminal law system.

## Discussion

The goal of the present study was to identify the prevalence of neurocognitive disorders in prisoners admitted to a PPC, and to describe the characteristics of this group. We found that 3.0% of the PPC population was diagnosed with a neurocognitive disorder. This is remarkably lower than the 20–23% found in two earlier studies in similar populations.^20,23^ Importantly, our study used real-world clinical data, whereas these two other studies used retrospective chart review to establish diagnoses of acquired brain injury themselves. A study by Fazel et al^26^ mentions that prevalence numbers of psychiatric disorders in prisons are also much lower in studies that use real-world clinical data, compared with studies that use structured instruments. Another remarkable finding of our study is the relatively high prevalence of an unspecified neurocognitive disorder (59.1% of the study sample). In sum, these findings lead us to hypothesise that neurocognitive disorders may be underdiagnosed in this population, and in light of the relatively high prevalence of unspecified neurocognitive disorder, this may be caused by a lack of expertise in neuropsychological assessment. In contrast, we should note that 42 of the individuals in this sample did not have a neurocognitive disorder at admission, but were diagnosed with one during their stay, indicating that at least in a small subgroup of patients, efforts were made to investigate the presence and nature of a neurocognitive disorder.

Another potential reason for a lack of sight on neurocognitive disorders in the practice of the PPCs may be that more than half of the sample was admitted because of aggressive or disturbing behaviour. In addition, we found high rates of comorbidity in this population. Our results show that only 24% of the study sample had no comorbid disorders, whereas in the control group, 42.7% had no comorbid disorders. In the study sample, substance misuse and addiction disorders (51.2%), psychotic disorders (20.9%) and personality disorders (23.2%) were common. One could imagine, with the main goal of the PPCs in mind (i.e. stabilisation), that the treatment staff focus on stabilising overt psychiatric conditions such as psychosis, severe mood disorders and severe aggressive behaviour and self-harm, thereby losing sight of the role that the neurocognitive disorder may play in these conditions and behaviours. However, knowledge of the nature of the neurocognitive disorder could actually be of benefit in this stabilisation phase. A great body of research has shown that the treatment of behavioural problems in dementia, for example, should be highly individualised after a thorough assessment,^27^ with an emphasis on specific non-pharmacological interventions. In addition, studies into the pharmacological treatment of agitation in patients with TBI show evidence for the use of propranolol, methylphenidate and valproic acid, besides the antipsychotic olanzapine.^28^ This may broaden the treatment options compared with the treatment of agitation within the context of, for example, a singular psychotic disorder. Interestingly, we found that individuals with a neurocognitive disorder had on average a 50-day longer stay in the PPC than the control group. We hypothesise that this may either be caused by a lack of the abovementioned specific treatment interventions in current clinical practice, or by the more chronic nature of neurocognitive disorders, and the extra complexity of the combination of neurocognitive disorders and comorbid psychiatric disorders.

Besides establishing a prevalence rate based on clinical data, this study sought to describe general and criminological characteristics of prisoners known to have a neurocognitive disorder. This group is characterised by low education levels (almost half did not have more than a primary education), an overrepresentation of individuals with a non-Dutch ethnicity (40.4%), a relatively high number of homeless individuals (26.4%) and a rather extensive history of mental healthcare, both in childhood (31.7%) and adulthood (72.1%). In addition, more than half of the group was diagnosed with substance misuse disorders, almost a quarter with a personality disorder and a fifth with a schizophrenia spectrum disorder. More than two-thirds of the sample was currently suspected of or convicted of a violent crime, and again two-thirds already had one or more convictions for a violent offense in the past. This indicates that this group has a large societal impact, especially when considering that half of the sample had had three or more convictions (for any type of crime) in the past 5 years.

Comparing these characteristics to data from the control group, we found that individuals with a neurocognitive disorder are 11.6 years older on average (48.5 *v.* 36.9 years), are significantly more often of a Dutch ethnicity (59.7% *v.* 34.4%) and have significantly more comorbid disorders (especially substance misuse and addiction disorders, 51.2% *v.* 41.7%). In addition, we found that our sample has a much lower prevalence of schizophrenia spectrum disorders (20.9% *v.* 57.5%). Our study sample also seems to have a different criminological profile: they are significantly more often repeat offenders (29.8% *v.* 17.5%) and commit significantly more property crimes (21.9% *v.* 14.5%) such as theft. Furthermore, they commit significantly less severe violent crimes, i.e. assault (4.8% *v.* 10.2%), although they commit murder significantly more often (10.4% *v.* 5.8%). Saliently, this leads to a combined percentage of 23.5% with convictions for manslaughter and murder in this sample.

The difference in mean age can most likely be explained by the increased odds of developing a neurocognitive disorder at an older age. The large difference in ethnicity may be caused by a higher degree of underdiagnosis of neurocognitive disorders in individuals with a non-Dutch ethnicity. Although there is increased attention for cross-cultural neuropsychological assessment in Europe in the past decades,^29^ there still is limited knowledge among clinicians on this topic (even among experts in neuropsychological assessment), and a limited availability of neuropsychological tests suitable for cross-cultural assessment.^29,30^

The difference in substance misuse disorders may be caused by neurocognitive disorders being a result of the substance misuse, although the lack of more specific diagnostic data prohibits us from testing this hypothesis. Simultaneously, it may also be possible that neurocognitive disorders lead to an increased risk of substance misuse, e.g. as a result of increased impulsivity. Indeed, in a longitudinal cohort study it was found that hospital admission because of TBI was positively associated with alcohol and drug dependence later in life.^5^

Finally, we see a much lower prevalence of schizophrenia spectrum disorders in our sample compared with the control group. We would argue that this implies that the behavioural problems that were a reason for admission, and that may be related to the neurocognitive disorder, are not likely to be mislabelled as psychotic behaviours in patients that are known to have a neurocognitive disorder. This lower prevalence also corroborates with the finding of Belfry et al,^23^ who reported lower prevalence of schizophrenia in forensic patients with acquired brain injury. The question remains, however, how these behavioural problems are interpreted in patients with an undiagnosed neurocognitive disorder.

Limitations of this study include the use of real-world clinical data, and the use of data specific to the PPCs. As mentioned earlier, real-world clinical data in prisons have shown to underestimate the true prevalence of disorders compared with the use of structured instruments in studies that systematically gathered data.^26^ The prevalence number of this study should therefore not be seen as a true prevalence rate, but rather the number of individuals that are identified as having a neurocognitive disorder in the current clinical practice. In addition, the characteristics that were found in this study may be different from those of currently undiagnosed individuals with a neurocognitive disorder. One may hypothesise that those who are currently diagnosed with a neurocognitive disorder are diagnosed because of certain apparent characteristics, e.g. the severity of their cognitive problems. Finally, using data from the PPCs results in a biased sample that is not representative of the total prison population in The Netherlands, and may also have poor international generalisability, as the handling of individuals with psychiatric disorders varies greatly between countries. In The Netherlands, prisoners with severe psychiatric problems are admitted to a psychiatric clinic within the prison system (the PPC), thus keeping the status of a prison or remand prison, whereas in many other countries, such patients are transferred to, for example, a forensic psychiatric hospital, a forensic wing in a regular psychiatric hospital or even a regular psychiatric hospital.

Future studies should aim to prospectively screen for neurocognitive disorders in forensic psychiatric patients, and follow up with a neurological and neuropsychological assessment when needed, to establish a true prevalence rate of neurocognitive disorders in this specific population. Subsequently, studies should focus on developing best practices for the treatment of these patients within a forensic context. Since we found a 50-day higher average length of stay in patients with a neurocognitive disorder, implying poorer treatment results or a need for more chronic care compared with other disorders, such best practices for the treatment of patients with a neurocognitive disorder in forensic care may lead to improvements in the functioning and quality of life of these patients, as well as significant cost reductions owing to a shorter length of stay. Although there are ample studies focusing on treatment within forensic psychiatry, there is an enormous gap in the literature regarding the treatment of neurocognitive disorders within this context. Investigating the efficacy and applicability of best practices from general (neuro)psychiatric facilities would probably be the best starting point for such intervention studies. In addition, it would be important for such research to identify complications for such best practices, such as a lack of neuropsychological expertise, or logistical difficulties that may arise when an increased number of forensic patients need to have magnetic resonance imaging scans at an external location.

In summary, although at face value our study suggests that only 3.0% of prisoners in a PPC have a neurocognitive disorder, we hypothesise that the true prevalence of neurocognitive disorders in this population is higher, based on findings in the literature^20,23^ and the relatively high prevalence of unspecified neurocognitive disorder. We call for increased attention to neurocognitive disorders in forensic psychiatry, both in clinical practice, as well as in research, including more attention for cross-cultural neuropsychological assessment.^29^ Neurocognitive disorders may play an important role in criminal offending, problematic behaviour and incidents within the prison system, as well as in the risk of reoffending. They should therefore be integrated in our everyday forensic psychologic and psychiatric practice and science, rather than be viewed as a separate or uncommon category of disorders.

## Data Availability

The data that support the findings of this study are not publicly available due to restrictions from the Custodial Institutions Agency. The data are available on request from the corresponding author, J.M., after formal approval from the regulatory body of the National Database of penitentiary psychiatric centres.

## Appendix A List of diagnostic codes included in this study

The following diagnostic codes were used to search for individuals with neurocognitive disorders in the National Database of penitentiary psychiatric centres.

**Table:**

DSM-5 diagnostic code	Disorder(s)
290.40	Major neurocognitive disorder possibly/probably due to vascular disease
291.1	Alcohol-induced major neurocognitive disorder, Amnestic confabulatory type
291.2	Alcohol-induced major neurocognitive disorder, Non-amnestic confabulatory type
291.89	Alcohol-induced mild neurocognitive disorder
292.82	Inhalant (major neurocognitive disorder), Sedative, hypnotic, or anxiolytic (major neurocognitive disorder), Other (or unknown) substance (major neurocognitive disorder)
292.89	Inhalant (mild neurocognitive disorder), Sedative, hypnotic, or anxiolytic (mild neurocognitive disorder), Other (or unknown) substance (mild neurocognitive disorder)
294.10	Major neurocognitive disorder due to … without behavioural disturbance
294.11	Major neurocognitive disorder due to … with behavioural disturbance
310.1	Personality change due to another medical condition
331.83	Mild neurocognitive disorder due to …
799.59	Unspecified neurocognitive disorder

**Table:**

DSM-IV-TR diagnostic code	Disorder(s)
290.40	Vascular dementia uncomplicated
290.41	Vascular dementia with delirium
290.42	Vascular dementia with delusions
290.43	Vascular dementia with depressed mood
291.1	Alcohol-induced persisting amnestic disorder
291.2	Alcohol-induced persisting dementia
292.82	Substance induced persistent dementia
292.83	Substance induced persisting amnestic disorder
294.0	Amnestic disorder due to [general medical condition]
294.10	Dementia due to … , without behavioural disturbance
294.11	Dementia due to … , with behavioural disturbance
294.8	Amnestic disorder NOS, dementia NOS
294.9	Cognitive disorder NOS
310.1	Personality change due to a general medical condition
780.93	Age-related cognitive decline

## References

[1] American Psychiatric Association. Diagnostic and Statistical Manual of Mental Disorders, Fifth Edition, Text Revision. American Psychiatric Publishing, 2022.

[2] Sachdev PS, Blacker D, Blazer DG, Ganguli M, Jeste DV, Paulsen JS, et al. Classifying neurocognitive disorders: the DSM-5 approach. Nat Rev Neurol 2014; 10: 634–42. 25266297 10.1038/nrneurol.2014.181

[3] Torrisi M, Cacciola A, Marra A, De Luca R, Bramanti P, Calabrò RS. Inappropriate behaviors and hypersexuality in individuals with dementia: an overview of a neglected issue. Geriatr Gerontol Int 2017; 17: 865–74. 27489168 10.1111/ggi.12854

[4] Cipriani G, Lucetti C, Danti S, Carlesi C, Nuti A. Violent and criminal manifestations in dementia patients. Geriatr Gerontol Int 2016; 16: 541–9. 26460091 10.1111/ggi.12608

[5] McKinlay A, Corrigan J, Horwood LJ, Fergusson DM. Substance abuse and criminal activities following traumatic brain injury in childhood, adolescence, and early adulthood. J Head Trauma Rehabil 2014; 29: 498–506. 24263173 10.1097/HTR.0000000000000001

[6] Bellesi G, Barker ED, Brown L, Valmaggia L. Pediatric traumatic brain injury and antisocial behavior: are they linked? A systematic review. Brain Inj 2019; 33: 1272–92. 31327257 10.1080/02699052.2019.1641621

[7] Fishbein D, Dariotis JK, Ferguson PL, Pickelsimer EE. Relationships between traumatic brain injury and illicit drug use and their association with aggression in inmates. Int J Offender Ther Comp Criminol 2016; 60: 575–97. 25326469 10.1177/0306624X14554778

[8] Vaughn MG, Salas-Wright CP, DeLisi M, Perron B. Correlates of traumatic brain injury among juvenile offenders: a multi-site study. Crim Behav Ment Heal 2014; 24: 188–203. 10.1002/cbm.1900 24425682

[9] Mendez MF. Behavioral variant frontotemporal dementia and social and criminal transgressions. J Neuropsychiatry Clin Neurosci 2022; 34: 328–40. 35306828 10.1176/appi.neuropsych.21080224

[10] Talaslahti T, Ginters M, Kautiainen H, Vataja R, Elonheimo H, Erkinjuntti T, et al. Criminal behavior in the four years preceding diagnosis of neurocognitive disorder: a nationwide register study in Finland. Am J Geriatr Psychiatry 2021; 29: 657–65. 33334647 10.1016/j.jagp.2020.11.011

[11] Ginters M, Talaslahti T, Palm A, Kautiainen H, Vataja R, Elonheimo H, et al. Criminal behaviour after diagnosis of a neurocognitive disorder: a nationwide Finnish register study. Am J Geriatr Psychiatry 2023; 31(8): 598–606. 36872165 10.1016/j.jagp.2023.01.025

[12] Shiroma EJ, Ferguson PL, Pickelsimer EE. Prevalence of traumatic brain injury in an offender population: a meta-analysis. J Correct Heal Care 2010; 16: 147–59. 10.1177/1078345809356538 20339132

[13] Farrer TJ, Hedges DW. Prevalence of traumatic brain injury in incarcerated groups compared to the general population: a meta-analysis. Prog Neuropsychopharmacology Biol Psychiatry 2011; 35: 390–4. 10.1016/j.pnpbp.2011.01.007 21238529

[14] Durand E, Chevignard M, Ruet A, Dereix A, Jourdan C, Pradat-Diehl P. History of traumatic brain injury in prison populations: a systematic review. Ann Phys Rehabil Med 2017; 60: 95–101. 28359842 10.1016/j.rehab.2017.02.003

[15] Allely CS. Prevalence and assessment of traumatic brain injury in prison inmates: a systematic PRISMA review. Brain Inj 2016; 30: 1161–80. 27484194 10.1080/02699052.2016.1191674

[16] Peacock S, Burles M, Hodson A, Kumaran M, MacRae R, Peternelj-Taylor C, et al. Older persons with dementia in prison: an integrative review. Int J Prison Health 2019; 16: 1–16. 32040274 10.1108/IJPH-01-2019-0007

[17] Brooke J, Diaz-Gil A, Jackson D. The impact of dementia in the prison setting: a systematic review. Dementia 2020; 19: 1509–31. 30269533 10.1177/1471301218801715

[18] Di Lorito C, Vӧllm B, Dening T. Psychiatric disorders among older prisoners: a systematic review and comparison study against older people in the community. Aging Ment Heal 2018; 22: 1–10. 10.1080/13607863.2017.1286453 28282734

[19] Coid J, Fazel S, Kahtan N. Elderly patients admitted to secure forensic psychiatry services. J Forensic Psychiatry 2002; 13: 416–27.

[20] Colantonio A, Stamenova V, Abramowitz C, Clarke D, Christensen B. Brain injury in a forensic psychiatry population. Brain Inj 2007; 21: 1353–60. 18066937 10.1080/02699050701785054

[21] Easden MH, Sakdalan JA. Clinical diagnostic features and dynamic risk factors in a New Zealand inpatient forensic mental health setting. Psychiatry, Psychol Law 2015; 22: 483–99.

[22] Witzel JG, Bogerts B, Schiltz K. Increased frequency of brain pathology in inmates of a high-security forensic institution: a qualitative CT and MRI scan study. Eur Arch Psychiatry Clin Neurosci 2016; 266: 533–41. 26174017 10.1007/s00406-015-0620-2

[23] Belfry KD, Ham E, Kolla NJ, Hilton NZ. Adverse childhood experiences and offending as a function of acquired brain injury among men in a high secure forensic psychiatric hospital. Can J Psychiatry 2023; 68: 453–60. 36537143 10.1177/07067437221144629 PMC10331256

[24] van Buitenen N, van den Berg C, Meijers J, Harte J. The prevalence of mental disorders and patterns of comorbidity within a large sample of mentally ill prisoners: a network analysis. Eur Psychiatry 2020; 63(1): e63. 10.1192/j.eurpsy.2020.63 PMC7355171 32522312

[25] Spreen M, Brand E, ter Horst P, Bogaerts S. *Historische, Klinische en Toekomstige – Revisie (HKT-R)*. Dr. van Mesdag kliniek, 2014.

[26] Fazel S, Hayes AJ, Bartellas K, Clerici M, Trestman R. Mental health of prisoners: prevalence, adverse outcomes, and interventions. Lancet Psychiatry 2016; 3: 871–81. 27426440 10.1016/S2215-0366(16)30142-0 PMC5008459

[27] Bessey LJ, Walaszek A. Management of behavioral and psychological symptoms of dementia. Curr Psychiatry Rep 2019; 21: 66. 31264056 10.1007/s11920-019-1049-5

[28] Williamson D, Frenette AJ, Burry LD, Perreault M, Charbonney E, Lamontagne F, et al. Pharmacological interventions for agitated behaviours in patients with traumatic brain injury: a systematic review. BMJ Open 2019; 9: e029604. 10.1136/bmjopen-2019-029604 PMC6615826 31289093

[29] Franzen S, Watermeyer TJ, Pomati S, Papma JM, Nielsen TR, Narme P, et al. Cross-cultural neuropsychological assessment in Europe: position statement of the European Consortium on Cross-cultural Neuropsychology (ECCroN). Clin Neuropsychol 2022; 36: 546–57. 34612169 10.1080/13854046.2021.1981456

[30] Franzen S, Papma JM, van den Berg E, Nielsen TR. Cross-cultural neuropsychological assessment in the European Union: a Delphi expert study. Arch Clin Neuropsychol 2021; 36: 815–30. 33043958 10.1093/arclin/acaa083 PMC8292927

